# A Novel Method to Model Image Creation Based on Mammographic Sensors Performance Parameters: A Theoretical Study

**DOI:** 10.3390/s23042335

**Published:** 2023-02-20

**Authors:** Nektarios Kalyvas, Anastasia Chamogeorgaki, Christos Michail, Aikaterini Skouroliakou, Panagiotis Liaparinos, Ioannis Valais, George Fountos, Ioannis Kandarakis

**Affiliations:** 1 Radiation Physics, Materials Technology and Biomedical Imaging Laboratory, Department of Biomedical Engineering, University of West Attica, 122 10 Athens, Greece; 2 Department of Biomedical Engineering, University of West Attica, 122 10 Athens, Greece

**Keywords:** mammography, Radeye sensor, digital phantom, simulated image, radiation detectors

## Abstract

Background: Mammographic digital imaging is based on X-ray sensors with solid image quality characteristics. These primarily include (a) a response curve that yields high contrast and image latitude, (b) a frequency response given by the Modulation Transfer Function (*MTF*), which enables small detail imaging and (c) the Normalize Noise Power Spectrum (*NNPS*) that shows the extent of the noise effect on image clarity. Methods: In this work, a methodological approach is introduced and described for creating digital phantom images based on the measured image quality properties of the sensor. For this purpose, a mathematical phantom, simulating breast tissue and lesions of blood, adipose, muscle, Ca and Ca(50%)-P(50%) was created by considering the corresponding X-ray attenuation coefficients. The simulated irradiation conditions of the phantom used four mammographic spectra assuming exponential attenuation. Published data regarding noise and blur of a commercial RadEye HR CMOS imaging sensor were used as input data for the resulting images. Results: It was found that the Ca and Ca(50%)-P(50%) lesions were visible in all exposure conditions. In addition, the W/Rh spectrum at 28 kVp provided more detailed images than the corresponding Mo/Mo spectrum. Conclusions: The presented methodology can act complementarily to image quality measurements, leading to initial optimization of the X-ray exposure parameters per clinical condition.

## 1. Introduction

Medical image diagnosis is based on accurately detecting suspicious lesions or regions in the vicinity of normal tissue. For this purpose, imaging modalities have been developed to highlight the tissue anatomical or functional images. One tissue with significant importance is breast tissue [1]. Worldwide, breast cancer is the second most commonly diagnosed cancer, accounting for around 11.6% of new cancer cases annually, and has a mortality rate of 6.6% [1,2]. An early breast cancer diagnosis can increase the probability of survival [3].

A prerequisite for early diagnosis is the use of an X-ray medical imaging detector and related X-ray imaging methodology [4]. The imaging methods currently in use are full-field digital mammography, digital tomosynthesis and contrast-enhanced mammography [4,5,6]. In addition, dual energy imaging methodologies in mammographic imaging with novel detectors have been reported [7,8]. X-ray detectors are evaluated either experimentally or by using theoretical models [9,10,11,12,13]. In each case, imaging metrics, such as the modulation transfer function (*MTF*) showing image sharpness, the normalized noise power spectrum (*NNPS*) showing image noise and the detective quantum efficiency (DQE) showing signal-to-noise transfer, are evaluated using standardized protocols [11,14,15,16,17,18]. This experimental procedure uses standardized X-ray beams [14,15,16], thus providing the basic significant characteristics of a sensor, such as the exposure range where the sensor is linear as well as its resolution and signal-to-noise transfer properties. The studies of this kind are powerful and objective tools and they are used to evaluate and compare imaging sensors performance.

In addition, physical phantoms may be used to determine other parameters such as contrast-to-noise ratio and detectability of lesions in noisy background [10,11,12,19,20,21,22]. This type of evaluation is important since the visibility of objects of different contrast and dimensions can lead to the optimization of the exposure and detector readout conditions per clinical examination, thus regulating the dose to the patient. However, the need for a physical observation of an image under specific exposure conditions, without actually exposing a phantom, has led to the development of the Monte Carlo code to simulate the effect of magnification in *MTF* [23], the development of breast phantom images [24] and the development of breast models that can be used for training [25,26].

In this work, an approach to exploit the knowledge of standardized sensor quality control data (e.g., *MTF* and *NNPS*) to simulate a digital image of a software phantom for digital breast projection imaging is attempted. The digital phantom is composed of simulated tissues through the related X-ray attenuation coefficients and assumed to be irradiated by four clinical mammographic X-ray spectra. Both low-contrast tissues and high-contrast materials for the energies under study have been considered. The structures have been designed with small dimensions to examine the contrast limits of the detector. For the image derivation, the published image quality parameters *MTF*, response curve and *NNPS* of a novel small-sized mammographic RadEye HR CMOS detector have been considered [27]. In this manner, a complementary understanding of the detector’s imaging capabilities in tissue imaging can be gained that can aid in the optimization of X-ray exposure conditions in terms of filter–target combination and incident dose to the patient.

## 2. Materials and Methods

### 2.1. Theoretical Image Generation

The detector considered was an indirect detection system with a 33.91 mg/cm^2^ phosphor coupled to a CMOS Remote RadEye HR photodiode pixel array of 1200 × 1600 pixels with pixel size of 22.5 μm. The image quality parameters of this detector (*MTF*, *NNPS* and DQE) were previously reported in the literature for an 8-bit mode of operation [27].

**Figure 1:**
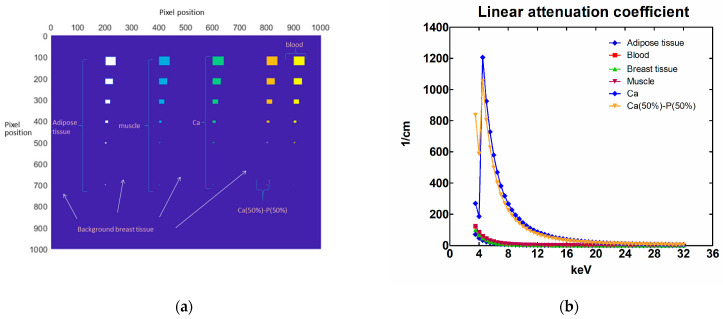
(**a**) The location of different lesion types on the mathematical phantom [30], and (**b**) the linear X-ray attenuation coefficients of the lesions (Reference 30 is licensed under CC BY-NC-SA 4.0).

In order to investigate the imaging performance of this detector, a 1000 × 1000 pixel subarray was created in Matlab [28]. In this array, rectangular areas of 2 × 2, 5 × 5, 10 × 10, 20 × 20, 30 × 30 and 40 × 40 matrix elements were taken. In these areas, the X-ray attenuation from adipose tissue (density 0.95 g/cm^3^), muscle (density 1.04 g/cm^3^), calcium (Ca) (density 1.55 g/cm^3^), a 50% calcium and 50% phosphorus mixture, Ca(50%)-P(50%) (density 1.82 g/cm^3^) and blood (density 1.06 g/cm^3^) was calculated by XmuDAt software (Version 1.0.1) [29]. The elemental thicknesses (t) were assumed to be 0.1 cm and 0.5 cm. The rest of the area was considered to be breast tissue (density 1.02 g/cm^3^) with a thickness of 4.2 cm and 6.0 cm. All the related X-ray attenuation coefficients have been calculated by multiplying the mass attenuation coefficients with the corresponding density, as obtained from the XmuDat software [29]. In Figure 1a, a graphical representation of the phantom with the available positions for the substances, as shown in the MATLAB environment, is demonstrated. The corresponding linear attenuation coefficients are shown in Figure 1b [29,30].

**Figure 2:**
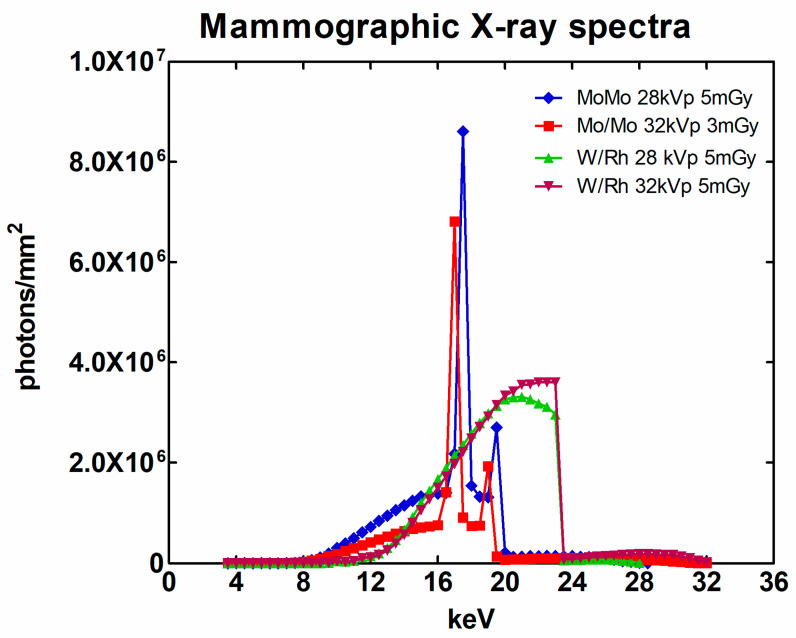
The mammographic X-ray spectra considered in this study.

The previously described software phantom was assumed to be irradiated by four mammographic X-ray spectra with anode/filter KVp combination as follows [31]: (a) Mo/Mo 28 kVp, (b) Mo/Mo 32 kVp, (c) W/Rh 28 kVp and (d) W/Rh 32 kVp. The Mo filtration was 0.03 mm and the Rh filtration was 0.06 mm [31]. The entrance surface air KERMA of the spectra was 5 mGy except in the case of Mo/Mo 32 kVp, which was 3 mGy. The mammographic spectra under consideration in this work are shown in Figure 2.

In order to calculate the incident exposure and subsequently the KERMA on the detector surface (the part of the X-ray spectrum that has propagated through the simulated phantom), we have employed an equation that considers that the X-ray spectrum [7,16] is exponentially attenuated in the tissues [31], as provided below:

(1) $$X_{T}=\sum_{E}1.83\cdot 10^{-6}\cdot f\left(E\right)\cdot e^{-\mu_{1}\left(E\right)T}\cdot E\cdot \frac{\mu }{\rho }{\left(E\right)}_{\mathrm{en},\mathrm{air}}$$

where *f*(*E*) is the X-ray fluence, *µ*_1_(*E*) is the X-ray attenuation coefficient of breast tissue for energy *E*, *T* is the breast thickness and (*µ*/ρ)*_en,air_* is the mass energy absorption coefficient of air. For the other substances with a thickness t, Equation (1) was adjusted as:

(2) $$X_{T,t}=\sum_{E}1.83\cdot 10^{-6}\cdot f\left(E\right)\cdot e^{-\mu_{1}\left(E\right)\left(T-t\right)}\cdot e^{-\mu_{2i}\left(E\right)t}\cdot E\cdot \frac{\mu }{\rho }{\left(E\right)}_{\mathrm{en},\mathrm{air}}$$

where *µ*_2_*_i_*(*E*) is the attenuation coefficient of tissue *i*, that is, blood, muscle, adipose, Ca and Ca(50%)-P(50%) tissue. The exposure that was calculated in this way in mR units, was turned into KERMA in mGy units, *K_T_* and *K_T,t_* by multiplying the exposures values *X_T_* and *X_T,t_* by 0.0087 [7,16], respectively. In this way, a KERMA image was imprinted on the 1000 × 1000 pixels.

Then, an initial assignment of signal values was implemented by employing the response curve of the detector *f*(*K*). The response curve, *f*(*K*) of the Radeye CMOS detector was obtained from the literature as f(K)=2.29·K−0.052 [27], where *K* is the KERMA incident on the detector surface.

The statistics of X-ray absorption and the presence of the detector introduced noise in the final image. This noise can be described by the *NNPS*, which is equal to NPS/M^2^, where *M* is the mean signal value and *NPS* is the noise power spectrum. It has been reported that the standard deviation (*SD*) may be approximated as [9] $\mathrm{SD}=\sqrt{\int \mathrm{NPS}\left(f\right)\mathrm{df}}$, where f is the spatial frequency. By considering the definition of *NNPS*, the coefficient of variation, *CV*, was calculated as follows:

(3) $$\mathrm{CV}=\sqrt{\frac{\int \mathrm{NPS}\left(f\right)\mathrm{df}}{M^{2}}}=\sqrt{\int \mathrm{NNPS}\left(f\right)\mathrm{df}\;}$$

In addition, by employing Equation (3) in the experimental published *NNPS* data [27], the *CV* was found to change with KERMA as [30]: $\mathrm{CV}=0.9256\cdot e^{-0.015\cdot K}$, R^2^ = 0.989.

For each pixel (*i,j*), the calculated KERMA, *K*(*i,j*), was applied to *f(Κ)* in order for the mean pixel value to be estimated. The standard deviation of the signal was found by multiplying the *CV* with *f*(*Κ*). In this way, a mean value and a standard deviation of the signal f(*K_T,t_*) and *SDK_T,t_* or f(*K_T_*) and *SDK_T_*, depending upon the existence of blood, muscle, adipose, or Ca and Ca(50%)-P(50%) tissues, could be obtained.

Assuming a normal distribution, the noise was inserted in the image through the “*normrnd*” MATLAB function [28], where the input parameters of “*normrnd*” are the mean value and the standard deviation.

The image creation procedure involves X-ray absorption in the image detector, creation of secondary quanta (electrons in direct conversion detectors or optical photons in indirect conversion detectors [9,13,17,31,32,33,34,35,36]), the spread of the secondary quanta to the output and the interaction in its electronic part. Therefore, the final image is characterized by visual unsharpness (i.e., blur) in object details and noise due to the statistical properties of signal propagation [9,10,11,12,13,14,31]. The blur can be described in the spatial frequency domain via the *MTF*. The *MTF* is considered ideal to be generated by a Point Spread Function (PSF) in the spatial domain where *MTF* is presented in the spatial frequency domain as the *Fourier* transform of the line *PSF* integral in one dimension:

(4) $$\mathrm{MTF}\left(f\right)={\mathrm{Fourier}}_{y}\left\{\int \mathrm{PSF}\left(x,y\right)\mathrm{dx}\right\}$$

Due to the spatial symmetry of the presented mathematical method, it is assumed that *MTF*(*f*) is equal in both the *x* and *y* axes. If *PSF*(*x*,*y*) is known then the blurred image can be calculated by a two-dimensional convolution of *PSF* with an image matrix.

The corresponding noise images were convolved with the detector *PSF* and an image was created where the signal *SV*(*i*,*j*) in each pixel (*i*,*j*) was affected by noise and blur.

If all the previous steps are considered, then an equation describing the addition of noise and blur in the image can be given as the following:

(5) SV(i,j)=PSF(i,j)∗normrnd(f[K(i,j)],{CV·f[K(i,j)})

where (∗) corresponds to convolution. The convolution was performed with the MATLAB ‘covn2’ function [28].

For reasons of better visualization, the calculated signal was linearly windowed in the range from 0 to 255 using the following formula:

(6) Im(i,j)=255SVmax−SVmin[SV(i,j)−SVmin] 

where *SV_min_* is the minimum *SV* value and *SV_max_* is the maximum.

**Figure 3:**
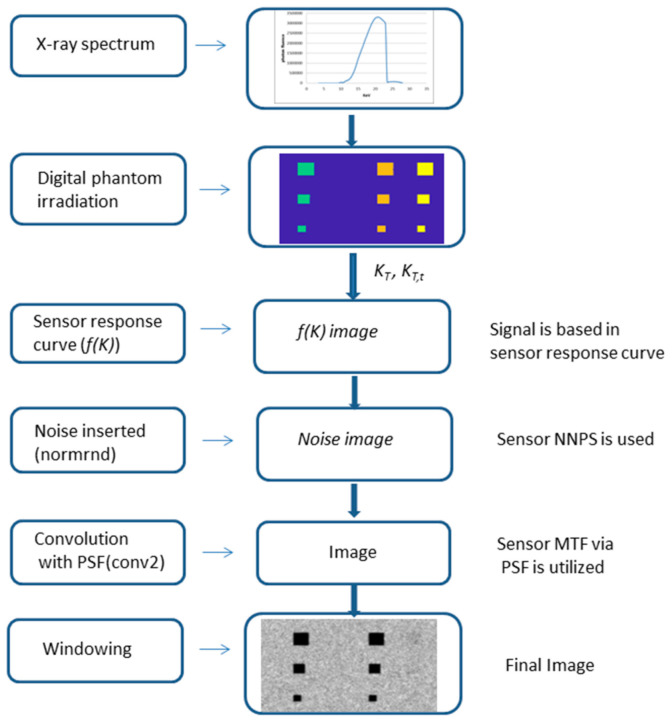
A flowchart of the method described in Equations (1) to (6).

In Figure 3, a flowchart demonstrating the procedure is shown.

### 2.2. PSF Estimation

It has been assumed that the *PSF* used in Equations (4) and (5) can be approximated by the rotation of a curve that can be described as the weighted sum of two Gaussian functions. The coefficients of the functions were obtained by trial and error, where for each coefficients combination, the *PSF* was obtained by rotating the resulted curve. Then a theoretical *MTF* was calculated based on Equation (4) and compared to the experimental one. The curve finally determined in this way is described by Equation (7) [30]:

(7) $$P\left(r\right)=0.6e^{-0.03r^{2}}+0.8e^{-0.1r^{2}}$$

**Figure 4:**
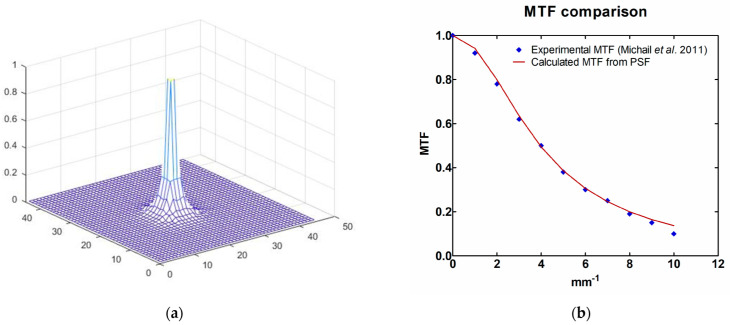
(**a**) *PSF* derived by rotating Equation (6), and (**b**) published experimental MTF results and the ones theoretically calculated by the *PSF* of this work [27,30] (Reference 30 is licensed under CC BY-NC-SA 4.0).

The *PSF*, calculated in this way with MATLAB software [26], is shown in Figure 4a and the comparison between the resulting *MTF*, derived by employing Equation (4), and the experimental one of the RadEye sensor, is shown in Figure 4b [27,30]. From Figure 4b, it can be seen that the *MTF* of the predicted value curves coincide well with the published experimental data, having a difference of 1.6% for spatial frequencies up to 9 mm^−1^. The calculated *MTF* curve has a higher value than the experimental one for 10 mm^−1^ spatial frequency, meaning that the true *PSF* may have a broader shape.

## 3. Results

### 3.1. Breast Size of 4.2 cm and Mo/Mo Spectra

**Figure 5:**
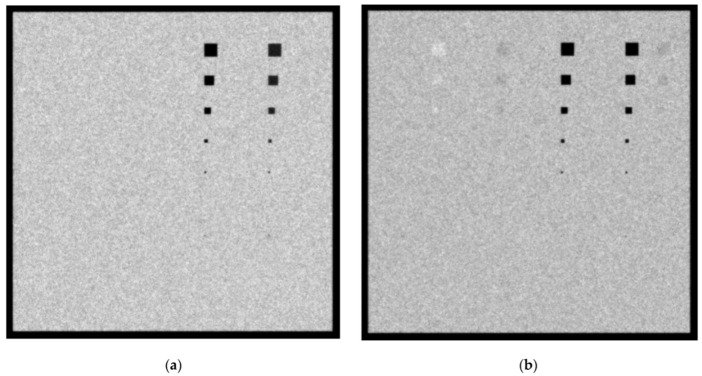
A 4.2 cm phantom image for Mo/Mo 5 mGy and 28 kVp spectrum with (**a**) 0.1 cm lesion thickness and (**b**) 0.5 cm lesion thickness.

**Figure 6:**
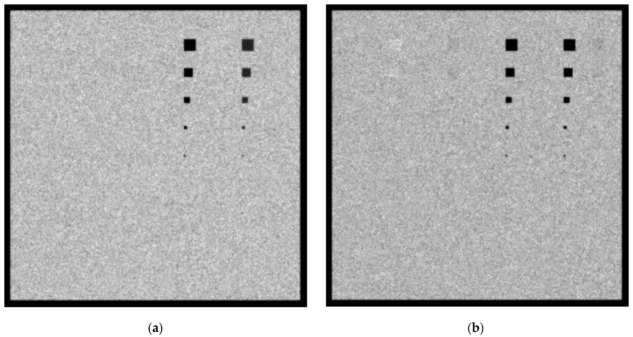
A 4.2 cm phantom image for Mo/Mo 3 mGy and 32 kVp spectrum with (**a**) 0.1 cm lesion thickness and (**b**) 0.5 cm lesion thickness.

In Figure 5a,b, the created images for the Mo/Mo 28 KVp and the 5 mGy incident spectrum with 4.2 cm phantom thickness for the 0.1 cm and 0.5 cm lesion thicknesses, respectively, are shown. When the 0.1 cm lesion thickness is considered, the Ca and Ca(50%)-P(50%) lesions up to 5 × 5 pixels (112.5 µm dimensions) are clearly visible. For the case of 0.5 cm-thickness lesions, shown in Figure 6b, the 20 × 20 pixels, corresponding to a size of 450 µm, can also be observed. A reason for the visualization of more materials for higher lesion thicknesses is the increase in subject contrast. As an example, for the 0.1 cm thickness, the X-ray contrast, *C_X_*, for adipose, muscle and blood was calculated with the formula *C_X_* = 100% (*K_T_* − *K_T_*_,*t*_)/*K_T_* and the values were approximately 1.9%, 1.9% and 2.2%, respectively. For the 0.5 cm thickness, *C_X_* was calculated as 10.8%, 9.2% and 10.3%, respectively. A smaller KERMA leads to a higher calculated *CV* value and a higher corresponding standard deviation used in the “*normrnd*” function.

In Figure 6a,b, the created images for the Mo/Mo 32 kVp and 3 mGy incident spectrum with a 4.2 cm phantom thickness for the 0.1 cm and the 0.5 cm lesion thicknesses, respectively, are shown. The Ca and Ca-P lesions, up to 5 × 5 pixels, are clearly visible and the smaller visible lesion is that of the 20 × 20 pixels. The *C_X_* values for adipose, muscle and blood at the 0.1 cm thickness were calculated as 1.8%, 1.9% and 2.8%, respectively. By contrast, for the 0.5 cm thickness, the corresponding *C_X_* values were 10.1%, 8.6% and 9.7%, respectively. The calculated *K_T_* for Mo/Mo 32 kVp 3mGy was 21.2 µGy, as compared to the 31.3 µGy calculated for the Mo/Mo 28 kVp 5mGy spectrum.

### 3.2. Breast Size of 4.2 cm and W/Rh Spectra 

**Figure 7:**
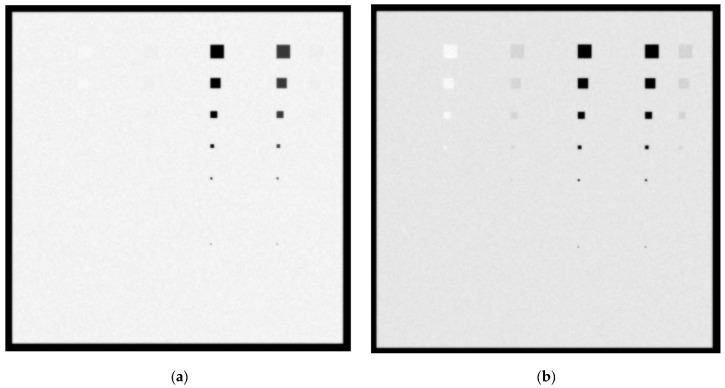
A 4.2 cm phantom image for W/Rh 5 mGy and 28 kVp spectrum with (**a**) 0.1 cm lesion thickness and (**b**) 0.5 cm lesion thickness.

In Figure 7a,b, the created images for the W/Rh 28 kVp and 5 mGy incident spectrum with a 4.2 cm phantom thickness for the 0.1 cm and 0.5 cm lesions thicknesses, respectively, are shown. When the 0.1 cm lesion thickness is considered, the Ca and Ca(50%)-P(50%) lesions up to a size of 5 × 5 pixels are visible. In addition, for adipose, muscle and blood tissues lesions, those as small as 20 × 20 pixels are roughly visible. When the 0.5 cm-thick lesions are considered, the 5 × 5 pixel lesion is visible in every case.

**Figure 8:**
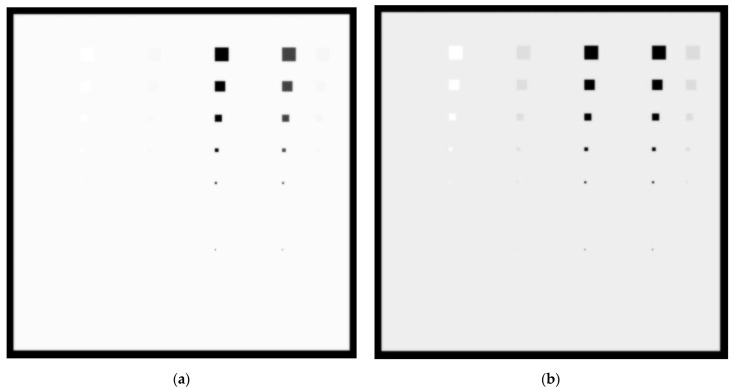
A 4.2 cm phantom image for W/Rh 5 mGy and 32 kVp spectrum with (**a**) 0.1 cm lesion thickness and (**b**) 0.5 cm lesion thickness.

In Figure 8a,b, the created images for the W/Rh 32 kVp and 5 mGy incident spectrum with 4.2 cm phantom thickness for 0.1 cm and 0.5 cm lesions thicknesses, respectively, are shown. When the 0.1 cm lesion thickness is considered, Ca and Ca(50%)-P(50%) lesions with sizes up to 2 × 2 pixels are visible. In addition, for adipose, muscle and blood tissues lesions, those as small as 20 × 20 pixels are roughly visible. When the 0.5 cm-thick lesions are considered, the 5 × 5 pixel lesion is visible in every case. A point worth noting is that the noise in Figure 7 and Figure 8 is lower than that of Figure 5 and Figure 6 due to the lower KERMA of the incident spectra, which yields a lower *CV* value for use in Equation (5).

### 3.3. Breast Size of 6.0 cm and Mo/Mo Spectra

**Figure 9:**
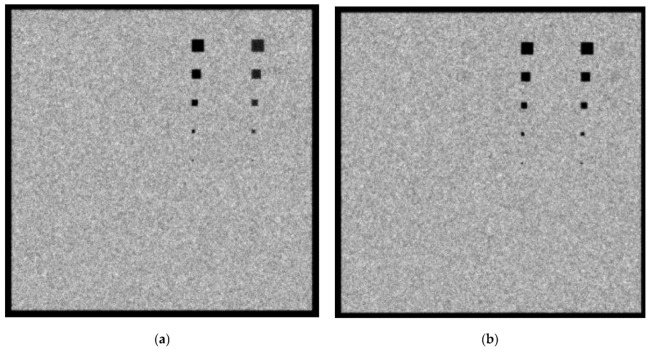
A 6 cm phantom image for Mo/Mo 5 mGy and 28 kVp spectrum with (**a**) 0.1 cm lesion thickness and (**b**) 0.5 cm lesion thickness.

In Figure 9a,b, the created images for the Mo/Mo 28 KVp and 5 mGy incident spectrum with a 6 cm phantom thickness for 0.1 cm and 0.5 cm lesions thicknesses, respectively, are shown. Ca and Ca(50%)-P(50%) lesions of up to 5 × 5 pixel size (112.5 µm) are visible. For the 0.5 cm thickness, blood lesions and adipose tissue sized up to 30 × 30 pixels (675 µm) may also be considered visible. The *C_X_* values for blood, adipose and muscle are 1.6%, 2% and 1.3%, respectively. The noise on the 6 cm tissue image is higher than the noise of the 4.2 cm image due to the higher X-ray absorption in the breast, leading to smaller KERMA values on the detector surface. The *K_T_* for the 6 cm breast is 7.5 µGy compared to 31.3 µGy for the case of the 4.2 cm breast irradiated with Mo/Mo 28 kVp and 5 mGy.

**Figure 10:**
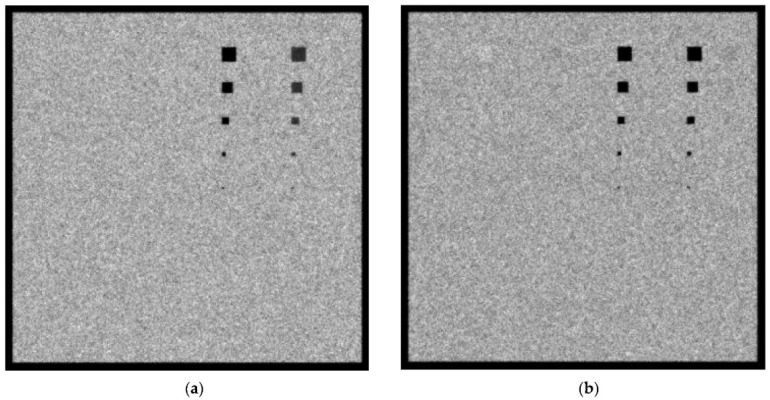
A 6 cm phantom image for Mo/Mo 3 mGy and 32 kVp spectrum with (**a**) 0.1 cm lesion thickness and (**b**) 0.5 cm lesion thickness.

In Figure 10a,b, the created images for the Mo/Mo 32 KVp and 3 mGy incident spectrum with a 6 cm phantom thickness for the 0.1 cm and 0.5 cm lesions thicknesses, respectively, are shown. Ca and Ca(50%)-P(50%) lesions up to 5 × 5 pixels in size (112.5 µm) are visible. For the 0.5 cm thickness, blood, muscle, and adipose tissue lesions of 40 × 40 pixels in size may also be considered visible, as well as blood lesions of 30 × 30 pixels in size. The *C_X_* values for blood, adipose and muscle are 4.5%, 1.7% and 4.2%, respectively. The low *K_T_*, that is equal to 5.7 µGy, introduces a higher image noise that prevents the clear visibility of smaller-sized lesions of the low-attenuation-coefficient materials.

### 3.4. Breast Size of 6.0 cm and W/Rh Spectra

**Figure 11:**
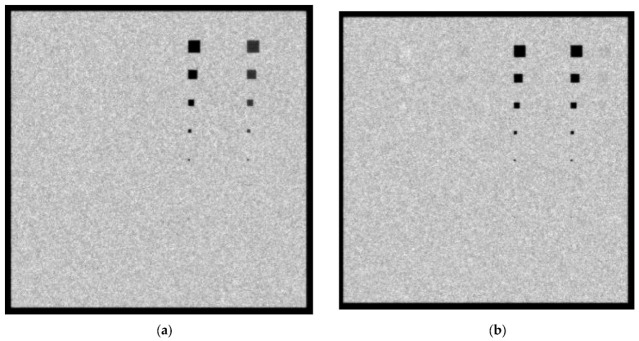
A 6 cm phantom image for W/Rh 5 mGy and 28 kVp spectrum with (**a**) 0.1 cm lesion thickness and (**b**) 0.5 cm lesion thickness.

In Figure 11a,b, the created images for the W/Rh 28 KVp and 5 mGy incident spectrum with a 6 cm phantom thickness for the 0.1 cm and 0.5 cm lesions thicknesses, respectively, are shown. For the 0.1 cm lesion thickness, only Ca and Ca(50%)-P(50%) lesions up to 5 × 5 pixels in size (112.5 µm) are visible. For the 0.5 cm thickness, blood, muscle and adipose tissue lesions up to 20 × 20 pixels in size may be considered visible. The *C_X_* values for blood, adipose and muscle at 0.5 cm are 8.1%, 6.7% and 6.9%, respectively. The incident KERMA on the detector was calculated to be equal to 28.25 µGy.

**Figure 12:**
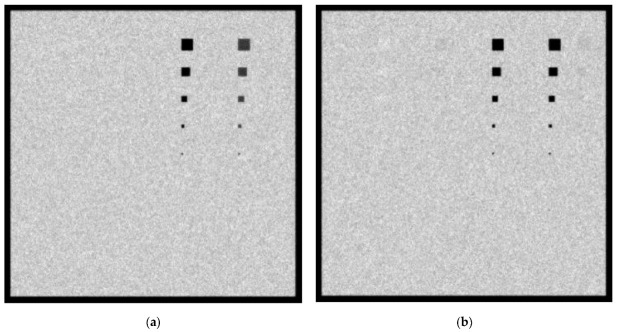
A 6 cm phantom image for W/Rh 5 mGy and 32 kVp spectrum with (**a**) 0.1 cm lesion thickness and (**b**) 0.5 cm lesion thickness.

In Figure 12a,b, the created images for the W/Rh 32 KVp and 5 mGy incident spectrum with a 6 cm phantom thickness for the 0.1 cm and 0.5 cm lesions thicknesses, respectively, are shown. Only Ca and Ca(50%)-P(50%) lesions up to 5 × 5 pixels in size are clearly visible. For the 0.5 cm thickness, however, muscle and adipose tissue can be seen up to 20 × 20 pixels in size. The *C_X_* values for blood, adipose and muscle at 0.5 cm are 7.1%, 6.9% and 5.7%, respectively. The incident KERMA on the detector was calculated as 34.35 µGy, resulting in a less noisy image in 6 cm breast tissue irradiation compared to the 28 kVp spectra.

In order to determine whether the low-contrast and not-visible regions of the images, (adipose, muscle and blood surrounded by tissue) contain information which might be extracted by image information enhancement algorithms, 10 program runs were performed, and inside each material lesion a concentric sub-region was selected and the average of the pixel values was calculated. The concentric sub-region for the 40 × 40 pixels, 20 × 20 pixels and 10 × 10 pixels comprised 625 pixels, 225 pixels and 49 pixels, respectively. The difference in average pixel values of the ROIs from the various structures and the background of the image were checked for normality (Kolmogorov—Smirnov) and then statistically tested with one-way ANOVA (a = 0.05). A Games–Howell post-hoc test was run on the data. The difference in average pixel values was considered statistically significant when *p* < 0.05. The ANOVA test was not performed for visible lesions. In addition, exposure conditions that are not usually employed in clinical practice (i.e., the 28 kVp Mo/Mo spectrum for 6 cm-thick breast tissue, or the 32 kVp W/Rh spectrum for 4.2 cm-thick breast tissue) were not considered in the test. Finally, from the remaining regions, only the larger non-visible region from each material was examined.

In Table 1, the results of the ANOVA test are demonstrated. As an initial choice, the large area lesions of 40 × 40 pixels were selected, since the lesions with larger areas are easier to distinguish [9,31,36]. It can be seen in Table 1 that, for the Mo/Mo 28 kVp irradiation conditions for the 4.2 cm breast thickness and the 0.1 cm lesion thickness, the adipose tissue is statistically comparable with the breast tissue background (*p* > 0.05), while for the blood and the muscle tissue, the signal is significantly different.

Additionally, when the Mo/Mo 32 kVp irradiation conditions are considered, all three tissues are statistically comparable with the breast tissue. For investigation purposes, the 0.5 cm-thick adipose lesion of 10 × 10 pixels in size, which is not visible under 28 kVp 5 mGy irradiation conditions, was examined and found to differ from the background (*p* = 0.004 < 0.05).

The 10 × 10-pixel low-attenuation lesions with a thickness of 0.1cm at 4.2 cm breast tissue irradiated with W/Rh spectra at 28 kVp 5 mGy, shown in Figure 9a, demonstrate statistical significance with the background tissue, implying that for 28 kVp the W/Rh spectrum is more efficient for imaging multiple low-contrast lesions than the Mo/Mo spectrum. For the 6 cm breast tissue size only, the W/Rh 32 kVp spectrum was examined. It was found that for the 0.1 cm lesion thickness, the adipose, muscle and blood tissue were comparable with the background, while for the 0.5 cm thickness lesion, the system can resolve the 20 × 20-pixel lesions, as shown in Figure 12a and Figure 12b, respectively.

## 4. Discussion

The presented theoretical results in Figure 6, Figure 7, Figure 8, Figure 9, Figure 10, Figure 11 and Figure 12 assume a linear response of the detector in every exposure condition under investigation, as well as in quantum limited exposure conditions. This is to say that the only noise present arises from the statistics of the X-rays and optical photon absorption and propagation in the RadEye HR detector. Our experimental data were under RQA-M2 exposure conditions, comprising a 28 kVp Mo/Mo spectrum with 2 mmAl added filtration and air kerma values on the detector surface of up to 40 µGy [27]. The RQA-M2 spectrum providing the experimental data had been experimentally measured by means of an Amptek spectrometer [27] and its mean energy was calculated as 18.8 keV. For the simulated spectra shown in Figure 5, the corresponding mean energies and KERMA incident on the detector r after passing through the 4.2 cm and 6 cm breast tissue are shown in Table 2.

The W/Rh spectra used to generate the images of Figure 8 and Figure 9 appear to be an optimum choice, primarily due to their low image noise. As previously mentioned, the *CV* value reduces exponentially with the incident KERMA on the detector. For the W/Rh 28 kVp 5 mGy, the *K_T_* was 92.2 µGy, and for the W/Rh 32 kVp 5 mGy; the corresponding KERMA value was 105.7 µGy. It should be noted, however, that the W/Rh 28 kVp 5 mGy spectrum yields a bit value of 211. This is very close to the maximum bit value allowed by the detector experiments. The same is true for the W/Rh 32 kVp spectra X-rays passing through 4.2 cm breast tissue, which produces a bit value of 242. Furthermore, the W/Rh 32 kVp spectrum is not usually a choice for 4.2 cm breast tissue. Therefore, despite the results of the mathematical simulation, only the Mo/Mo 28 kVp 5mGy and Mo/Mo 32 kVp 3 mGy may provide realistic results for the 4.2 cm breast thickness. For the W/Rh 28 kVp 5 mGy and 32 kVp 5 mGy spectra, although they produce pixel values within the 8-bit range available from our experiments, there is no information regarding whether they are in the linear detector range or not.

If the 6 cm breast thickness is considered, all the exposure values are within the detector linear range (i.e., below 40 µGy) [27]. However, the W/Rh 32 kVp spectrum is more likely to be employed in a larger breast examination than the 28 kVp spectra.

Furthermore, the object visibility is a function of contrast, noise and lesion size [17,30,35]. As seen in Table 1, for the 4.2 cm breast and 0.1 cm lesion thicknesses, at the exposure conditions within the experimentally determined linear detector exposure range (6–40 µGy) [27], only the muscle tissue signal retains information that may be extracted. For the corresponding 0.5 cm thickness, lesion sizes of 225 μm (10 pixels) can be resolved.

Finally, in almost all of the images, the high-contrast lesions (Ca and Ca(50%)-P(50%)) are visible even for the 2 × 2-pixel lesions. Despite the fact that our applied *PSF* may overestimate the response at high frequencies, as shown from the derived *MTF* value at a frequency of 10 mm^−1^ in Figure 4b, it is anticipated that the high *C_X_* values of Ca and Ca(50%)-P(50%), over 70.8% in each case, could employ sufficient signal difference with respect to the background.

The presented method does not consider the scatter of the X-ray photons in the tissue mass [10]. However, if we assume that a good grid eliminates more than 80% of the scatter radiation and allows the transmittance of at least 75% of the useful beam [37], our results may be considered equivalent to those derived from 4 mGy (otherwise 3/0.75 mGy and 6.67 mGy otherwise 5/0.75 mGy irradiation spectra), with a grid of 75% transmittance in the beam. Nevertheless, the use of the measured *MTF* and *NNPS* values incorporates all X-ray scatter phenomena that the X-rays might be subjected to in the detector. Thus, the derived image is an ideal tissue image that a detector with the specific experimentally calculated image quality parameters could yield. It may provide information for the minimum lesion dimension that can be imaged for various X-ray spectra and KERMA combinations. Hence, it acts complementarily to the image quality measurements of the detector.

Besides the examined detector in the mammographic range, the method can be applied to every detector type, provided the basic image quality parameters such as *MTF*, *NNPS* and response function are available. It may be used as a first step for X-ray exposure optimization by modelling different breast sizes with lesions of different dimensions that are irradiated with different exposure conditions in terms of filter–target combination, kVp and KERMA. The resulting images may be of service as an element in the optimization of exposure conditions, as well as the development and study of software algorithms that enhance lesion identification and detectability [38,39,40].

## 5. Conclusions

In the present study, a method for the generation of simulated phantom images, based on specific detector image quality characteristics, is described. The method has been applied in a RadEye HR digital high-resolution detector via the design of a mathematical phantom. The method was used to examine the eligibility of four mammographic X-ray spectra and discover the extent of the detector’s imaging capabilities for lesion thicknesses between 0.1 cm and 0.5 cm and areas between 2 × 2 pixels and 40 × 40 pixels, where 1 pixel equals 22.5 µm. The presented method can be applied to every detector where the *MTF*, *NNPS* and response function curves are available. It may be utilized complementarily to *MTF* and *NNPS* parameters for X-ray exposure optimization and to serve as a baseline for studying the applicability of various software algorithms that enhance lesion identification and detectability.

## Figures and Tables

**Table 1:** Results for ANOVA test for the low-contrast objects under investigation with respect to the background.

Irradiation Conditions	Breast Thickness/Lesion Size/Lesion Thickness	Figure	Adipose	Blood	Muscle
Mo/Mo, 28 kVp, 5 mGy	4.2 cm/40 × 40/0.1 cm	5a	*p* = 0.568	*p* = 0.01	*p* = 0.039
Mo/Mo, 32 kVp, 3 mGy	4.2 cm/40 × 40/0.1 cm	6a	*p* = 0.757	*p* = 0.107	*p* = 0.346
Mo/Mo, 28 kVp, 5 mGy	4.2 cm/10 × 10/0.5 cm	7b	*p* = 0.004	-------	--------
W/Rh, 28 kVp, 5 mGy	4.2 cm 10 × 10/0.1 cm	8a	*p* = 0.002	*p* < 0.001	*p* < 0.001
W/Rh, 32 kVp, 5 mGy	6 cm/40 × 40/0.1 cm	12a	*p* = 0.848	*p* = 0.544	*p* = 0.057
W/Rh, 32 kVp, 5 mGy	6 cm/20 × 20/0.5 cm	12b	*p* < 0.001	*p* < 0.001	*p* = 0.006

**Table 2:** The mean energy of the X-ray photon spectra and the Kerma on the detector surface for the breast thicknesses of 4.2 cm and 6 cm used in this study.

Irradiation Conditions	Breast Thickness (cm)	E_mean_ on Detector Surface (keV)	KERMA on Detector Surface (µGy)
Mo/Mo, 28 kVp, 5 mGy	4.2	19.90	31.30
Mo/Mo, 32 kVp, 3 mGy	4.2	22.08	21.20
W/Rh, 28 kVp, 5 mGy	4.2	21.15	92.20
W/Rh, 32 kVp, 5 mGy	4.2	22.18	105.70
Mo/Mo, 28 kVp, 5 mGy	6	21.15	7.50
Mo/Mo, 32 kVp, 3 mGy	6	24.07	5.70
W/Rh, 28 kVp, 5 mGy	6	21.54	28.25
W/Rh, 32 kVp, 5 mGy	6	22.93	34.35

## Data Availability

Data are contained within the article.
